# Malignancy and Endocarditis: Divulging Into the Intertwined Association

**DOI:** 10.7759/cureus.24089

**Published:** 2022-04-12

**Authors:** Lakshmi Sree Pugalenthi, Mahlika Ahmad, Sanjana Reddy, Zineb Barkhane, Jalal Elmadi, Lakshmi Satish Kumar

**Affiliations:** 1 Internal Medicine, Ago Medical and Educational Center Bicol Christian College of Medicine, Legazpi City, PHL; 2 Research, Ziauddin University, Karachi, PAK; 3 Medicine, Bogomolets National Medical University, Kiev, UKR; 4 Research, Universite Hassan II Faculté de Médecine et de Pharmacie de Casablanca, Casablanca, MAR; 5 Facultad de Ciencias Médicas, Universidad Nacional Autónoma de Honduras, Tegucigalpa, HND; 6 Internal Medicine, University of Perpetual Help System DALTA, Manila, PHL

**Keywords:** colorectal cancer and endocarditis, malignancy and endocarditis, streptococcus bovis endocarditis, s. anginosus endocarditis, marantic endocarditis, non-bacterial thrombotic endocarditis, infective endocarditis

## Abstract

Cancer is an immunosuppressive disorder with characteristic features of unchecked cell growth, invasion, and sometimes thromboembolism leading to multiple systemic sequelae, including infective endocarditis. This article has compiled some of the crucial mechanisms by which infective endocarditis occurs in cancer patients, its risk factors, and the existing treatment interventions. It has focused on the necessity of being aware that these multiple pathogeneses are involved in the development of infective endocarditis (IE) in cancer patients, which would help delineate the risk factors associated with the condition and help physicians screen better for specific red flags. Identifying these risk factors and patient-oriented therapy, targeting the necessary elements such as causative organism, patient immune status, type of cancer, choosing evidence-based treatment modalities, and to improve the outcome of the disease in an already exasperating condition called cancer.

## Introduction and background

Cancer is the uncontrolled growth of a cell with the subsequent development of metastatic features. Malignancy is when the unchecked cell growth develops into a metastatic state by downregulation of cell adhesion receptors and up-regulation of receptors for increased cell motility for its spread far from its origin [1]. Cancer patients with infective endocarditis (IE) might be active or previously diagnosed. IE can also be a marker for the suspicion of an underlying malignancy [2,3]. One of the first associations between IE and malignancy was described decades ago in patients with colorectal cancer (CRC) [3]. The occurrence of IE in cancer patients is surprisingly uncommon and was found to be 18% [4]. The most common cancers in the United States and Europe are colorectal, rectal, breast, and prostate, with a collective incidence of 800,000 and 1,370,000, respectively [5,6]. A recent study identified that IE occurred more commonly in patients with lung, prostate, and colorectal cancers than in patients without a cancer diagnosis [3]. Other extensive databases have observed that some digestive, respiratory, and hematologic malignancies are related to IE [7]. IE cancer patients belonged to the elderly population and were most often males [4,8,9].

Cancer patients undergo a substantial amount of invasive procedures during diagnosis and treatment, which puts them at risk of developing IE [10]. The thrombotic nature of most malignancies is one of the most well-discussed mechanisms of IE in patients with cancer [11]. In recent times, technological advancement has shed some light on the relatively less known mechanism by which malignancies serve as a threat to the development of IE, such as acting as a port of entry (POE) via the skin, oral, biliary, urinary, female genital tract [12] or by the associated immune suppression. Certain bacteria can adhere and invade tissues as another crucial etiology of IE in cancer patients [7]. Hematologic malignancies and specific chemotherapeutic treatments are prone to cause immunosuppression, which acts as a segue for IE development [13]. Compared to classic IE in non-cancer patients, IE in cancer patients present atypically with significantly less fever and a new heart murmur [14]. The complications of cancer with subsequent IE have a slightly different and increased preference for acute renal failure, followed by emboli events and congestive heart failure (CHF) [14].

Positron emission tomography with 2-deoxy-2-fluorine-18 fluoro- D-glucose integrated with computed tomography ((18F-FDG) PET/CT) has a significant diagnostic value in cancer and non-cancer patients. However, it has some drawbacks, such as the inability to differentiate between metastatic lesions, inflammatory foci, and emboli of IE. Although surgical indications are similar to non-cancer patients, cardiac surgeries are not often performed. The cancer patients with IE were denied surgery mainly because of their high-risk features, and delaying the surgery may lead to death before performing the procedure itself [14].

There remain unfamiliar grounds for the disease characteristics and risk factors of IE that could occur in patients with an underlying malignancy, which would be helpful to understand the disease course better and provide appropriate treatment [15]. This article aims to discuss the pathophysiology of IE in patients diagnosed with malignancy and its risk factors, making the patient susceptible to the simultaneous diagnosis, and highlighting the treatment modalities available for IE in cancer patients.

## Review

Pathogenesis

**Figure 1:**
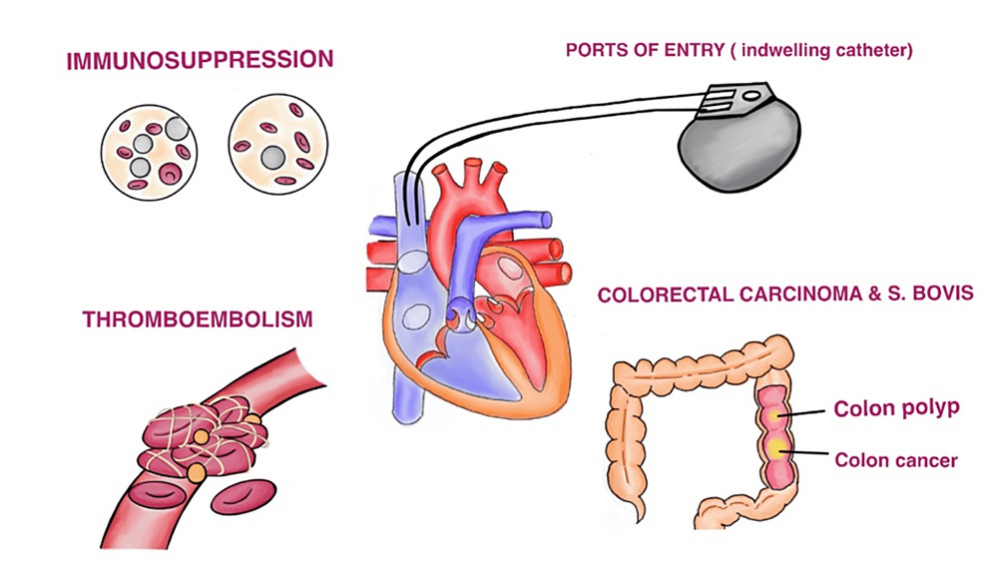
Pathogenesis of infective endocarditis in cancer patients Image credits - Lakshmi Sree Pugalenthi S. bovis  - Streptococcus bovis

As discussed earlier, IE can originate from various sources in cancer patients, as depicted in Figure 1.

Immunosuppression

Immune suppression plays a vital role in the pathophysiology of endocarditis in cancer patients. Immune suppression has various etiologies in such patients, such as chemotherapy, alcoholism, metastatic disease, and old age [1,3,12,16,17]. Patients with hematologic malignancies have neutropenia associated with poor phagocytosis, abnormal B & T cells, POE from venous catheter routes, splenectomy, and chemotherapy-related immune suppression. These are some of the suggested conditions responsible for the development of endocarditis in cancer patients [7].

Cancer patients are prone to be neutropenic, making them susceptible to IE. Cristina et al. conducted a study over 10 years, from 2005 to 2015, in Santa Cruz de Tenerife. The study population included 208 IE patients, of which 32 people were also diagnosed with cancer. The study concluded that neutropenia was detected during the time of the diagnosis of IE in only 15.6% of the patients [18]. Chemotherapeutic agents inhibit the synthesis of DNA and RNA, which results in the suppressed production of immune cells [19]. Cancer patients have a poor nutritional status which further impairs the B & T cells' immuno-protective effects [19].

*S. bovis* endocarditis and CRC

The association between CRC and Streptococcal endocarditis was discussed in 1951 [3]. The strain, *Streptococcus bovis*, seemed to have an undeniable relationship with colon cancer [20]. *S. bovis*-associated endocarditis has an unusual and slightly less understood relationship with colorectal carcinoma. *S. bovis* is a commensal bacteria inhabiting the lumens of our gastrointestinal system and is responsible for 10-15% of all IE cases [21,22]. Any colonic lesion may serve as a niche for the colonization of *S. bovis*/*gallolyticus*. Hence premalignant colonic lesions serve as a site of colonization for *S. bovis*. The ability of the bacteria to survive for a more extended period is increased due to the conducive environment provided by the tumor microenvironment [23]. Once intestinal micro-perforations occur in CRC, these colonized organisms find their way into the blood circulation [21,22].

The diagnosis of advanced adenoma or cancer with *S. bovis* colonization is made before the development of endocarditis has been found in many cases [24]. *S. bovis* has characteristic traits such as adhesion to intestinal cells by pili expression due to its interaction with mucin and collagen [25,26] and the ability to grow in bile compared to other alpha-hemolytic streptococci. Due to these properties, *S. bovis* can bypass the hepatic-reticuloendothelial system and enter the systemic circulation directly [27,28]. 

*S. bovis* has also been known to play a role in the development of CRC actively, explained by an increased interleukin 1 (IL-1), cyclooxygenase-2 (COX-2), and interleukin-8 (IL-8). These cytokines stage an inflammatory sequence for tumor development and progression [29]. The permeability of the intestinal wall is further altered by the production of cytokines by the bacteria itself, promoting its entry from the intestinal lumen into the blood, resulting in a myriad of infectious complications [30].

Port of entry

As the name suggests, POE serves as a port for bacterial organisms to cause bacteremia and subsequently IE. Bacterial characteristics such as pili [31,32], biofilm formation for adhesion to epithelial cells [33-37], the ability to attach to the extracellular matrix of the heart valves [33,34,36,37], and its capacity to evade phagocytosis [35] and thereby aiding in bacterial virulence. The most common POEs are the skin, oral cavity, and digestive system [38]. Identifying possible POE is crucial in reducing the incidence of a new episode of IE. The various POE and the associated risk factors are discussed in Table 1 [38].

**Table 1:** Ports of entry and their risk factors POE - Port of Entry IV - Intravenous BPH - Benign Prostatic Hyperplasia ENT - Ear, nose & throat

POE	RISK FACTORS
Cutaneous	Healthcare-associated Vascular access, infection of cardiac implantable electronic device, infection of the operation site, community-acquired {domestic wound, ulcers (diabetic foot ulcer, venous ulcer, pressure ulcer), insect bite}, IV drug use, inoculation disease (louse bite, tick bite, cat-scratch disease).
Oral/ dental infective foci	Somatologic examination, dental infectious focus, and endodontic & periodontal disease.
Colonic Lesions	Those who underwent colonoscopy for polyps, diverticulosis, diffuse angiodysplasia, and adenocarcinoma.
Urinary Lesions	Who underwent urinary examinations: Prostate cancer, BPH with urine retention, urethral stenosis, pyelonephritis, cystinuria with repetitive renal lithiasis, post-radiotherapy bladder, and extrinsic urethral compression by colon cancer.
ENT lesions	Sinusitis, otomastoiditis, etc.

Delahaye et al. conducted a study in France over six years in a sample population of 318 hospitalized patients, excluding 82 patients who died during the hospitalization. They found that 78% of the patients were identified with POE. Cutaneous POE (40%) was the most commonly identified source, followed by 29% and 24% in dental/oral POE and gastrointestinal POE, respectively. The study concluded that POE is directly associated with a diagnosis of IE and that patients with such possible POE must be examined regularly [38].

Invasive procedures pose a risk for the development of IE. Kim et al. conducted a study from 2011 to 2015 on 170 patients, of which 30 patients had active cancer. It was found that non-dental procedures can also lead to IE, such as intravenous catheter insertion and endoscopic or genitourinary invasive procedures [18].

Non-bacterial thrombotic endocarditis

Non-bacterial thrombotic endocarditis (NBTE) is common among those between 40 to 80 years of age, with no preference for a specific gender. Adenocarcinoma is a malignancy that is most commonly associated with NBTE, especially in the lungs, pancreas, and gastric adenocarcinomas [39].

Many studies have estimated that 15% of cancer patients have developed a thromboembolic event throughout their disease course and 50% have a documented venous thromboembolic event [40,41]. NBTE is characterized by the presence of vegetations on the heart valves, which are sterile and non-infective [42]. Cancers are associated with the increased secretion of cytokines such as tumor necrosis factor (TNF) or IL-1, which causes local tissue damage; and subsequently, vegetation formation [43]. NBTE is based on the mechanism of endothelial cell injury with a coexisting hypercoagulable state. NBTE is found to have increased levels of circulating tissue factors. The monocytes on valvular lesions express more tissue factor messenger RNA (mRNA), which contributes to the mechanism of coagulation cascade initiation [44]. This injury leads to thrombi formed by platelet accumulation and inflammatory mononuclear cells entangled with fibrin and immune complexes [45]. Cardiac lesions in such a setting are always devoid of microorganisms making NBTE distinct from IE. They break apart more easily and can seed out into the systemic circulation. NBTE vegetative lesions can equally affect damaged and undamaged valves, chordae tendineae, or the endocardium [45]. Histologically, these vegetations seem to show some evidence of abnormal collagen and elastic fibers. Valvular leaflets with areas of high flow seem to influence the development of valvular lesions associated with NBTE [43]. The interaction between macrophages and malignant cells leads to the release of cytokines such as TNF, IL-1, and IL-6, promoting endothelium damage, platelet deposition, and the formation of friable thrombi. Such interactions also amplify the coagulation cascade, resulting in a worsened hypercoagulable state [46]. These patients become clinically symptomatic only when the thrombus has embolized to the brain or any other vital organ [38]. Recent studies have found a link between oncogene and activation and hemostasis, whereby the mesenchymal-epithelial transition factor (MET) oncogene causes transcriptional response and upregulation of plasminogen activator inhibitor type 1 (PAI-1) and cyclooxygenase 2 (COX-2), ultimately resulting in increased coagulation [47].

Treatment modalities and outcomes

Mistiaen and Gebruers conducted a literature search from 2010 to 2018 focusing on IE and malignancy. It was found that the short-term outcome of IE was not altered significantly by malignancy, and no particular pattern of management strategy was observed [7].

Del Castillo-Payá conducted a retrospective observational study from 2005 to 2015 in 208 IE patients, of which 32 patients were also diagnosed with cancer. They concluded that the Charlson comorbidity index remained unchanged irrespective of whether the patient was diagnosed with cancer or not. Surgery was not performed in 18.7% of the patients even though there was an indication [48]. The in-hospital mortality and survival rate is comparatively higher in cancer patients with IE than in non-cancer patients with IE (Table 2); and hence, a worse outcome.

**Table 2:** Outcomes of patients with IE and cancer IE - Infective Endocarditis

REFERENCE	TIMELINE OF STUDY	STUDY TYPE	POPULATION	CONCLUSION
Cullen Grable, et al. (2021) [49].	2001-2006 and 2015-2018	Retrospective	56 patients with the diagnosis of cancer and IE	Cancer IE patients had a poorer survival rate than those in remission (HR 2.497; 95% CI 1.062 to 5.868; p=0.0358).
Bernard Cosyns, et al. (2020) [50].	2016-2018	Prospective cohort study	3085 IE patients (359 cancer IE patients, 2726 cancer free IE patients) from 40 countries	Higher in-hospital mortality in cancer IE patients (23.4 vs. 16.1%, P = 0.006, and 18.0 vs. 10.2%; P < 0.001, respectively.)
Del Castillo et al. (2018) [48]	2005 to 2015	Retrospective observational study	208 IE patients, of which 32 patients also had cancer	In-hospital mortality was 45.5%, and the probability of survival was 40%
Kyu Kim, et al. (2017) [18]	2011 to 2015	Retrospective cohort study	170 patients with IE	In-hospital mortality was higher in patients with an additional diagnosis (34.4% vs. 12.4%, P<0.001).
Ana Fernández-Cruz, et al. (2017) [8].	2008 to 2014	Prospectively included	161 patients diagnosed with IE in 30 hospitals	Cancer patients with IE had higher In-hospital mortality (34.8% vs. 25.8%, P = .012) and 1-year mortality (47.8% vs. 30.9%, P

**Figure 2:**
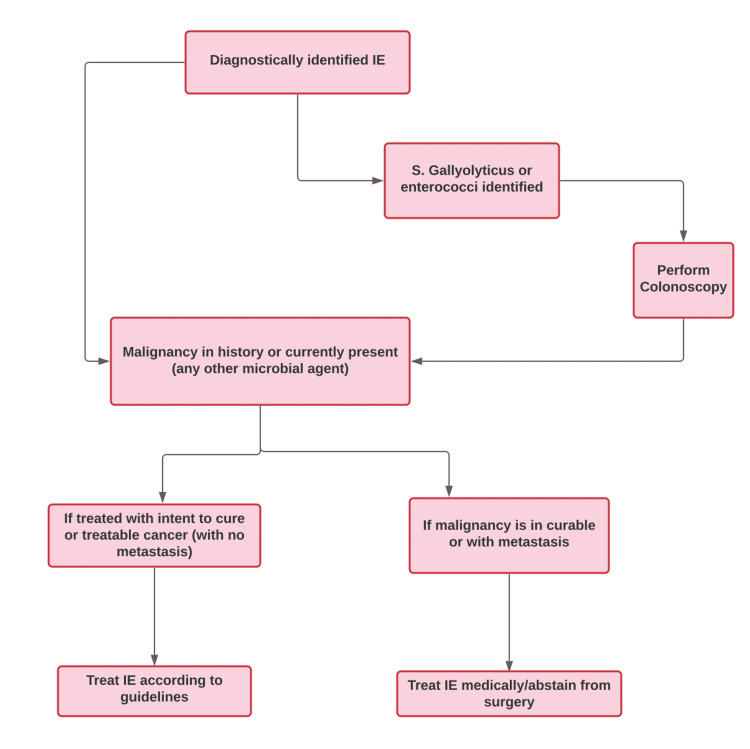
Proposed treatment guidelines for infective endocarditis in cancer patients Image credits - Lakshmi Sree Pugalenthi IE - Infective Endocarditis S. gallolyticus - Streptococcus gallolyticus

The goal of treating IE in cancer patients should be to (1) prevent bacteremia, (2) consider fever as a warning sign, and (3) rule out an alternate diagnosis to endocarditis [48]. It is suggested that patients with a treatable malignancy and concomitant IE should be prioritized and be treated for IE according to available guidelines [7]. A proposed treatment guideline for IE in cancer patients is depicted in Figure 2. 

Medical intervention

Empiric antimicrobial treatment depends on various factors such as microorganisms suspected and the location of the cardiac lesion [51,52]. *Staphylococcus aureus* must always be a suspected pathogen in right-sided native valve endocarditis (NVE). Antibiotic therapy must be targeted with *S. aureus* coverage, such as penicillinase-resistant penicillin or vancomycin, based on the prevalence of methicillin-resistant *S. aureus* (MRSA) in the locality [53,54].

Trends are slightly starting to change in recent years with the empiric treatment. Cosyns et al. conducted a study across 40 countries from 2016 to 2018 with 3085 patients diagnosed with IE. They reported that IE-cancer patients were more frequently treated with amoxicillin and daptomycin than with vancomycin [49].

Although invasive procedures serve as POE in cancer patients, the effects of antibiotic prophylaxis on such patients are still being studied. Since patients with cancer are not typically considered at risk of developing IE, antibiotic prophylaxis is not currently a recommendation [18]. Although still under evaluation, antibiotic prophylaxis has not shown significant results in preventing increased infection rates associated with invasive procedures in critical patients (Table 3).

**Table 3:** Outcomes of antibiotic prophylaxis as per included studies CRI - Catheter-Related Infections CVAP - Central Venous Access Ports

Reference	Design	Cases	Conclusion
Evan Johnson et al. (2016) [55]	Meta-analysis	2154	No significant difference in infection rates.
Anne M. Covey et al. (2012) [56]	Retrospective chart review	1183	The infection rate without antibiotic prophylaxis is < 1%.
Courtney L Scaife et al. (2010) [57]	Retrospective review	459	All 9 (2%) CRIs occurred in the non-prophylactic antibiotic group (P = .218), with 5 infections resulting in port removal. Single-dose perioperative antibiotics may decrease CVAP infection rates and should be studied further in a prospective randomized trial.

Patients with malignancy-associated NBTE were found to have a poor long-term outcome. Still, such patients were treated with anticoagulant therapies with significant palliative effects [43]. Treatment in NBTE aims to control underlying cancer and its instigating factor, thromboembolism, with or without associated disseminated intravascular coagulation (DIC) [58]. The American Association of Colleges of Pharmacy (AACP) guidelines have recommended that patients with NBTE and systemic/pulmonary emboli be given full-dose intravenous unfractionated heparin or subcutaneous low molecular weight heparin, and patients with disseminated cancer with aseptic vegetations should be treated with full-dose intravenous unfractionated heparin or subcutaneous low molecular weight heparin [59].

Surgical management

The main goal of surgical intervention in IE is to remove the infected tissue, reduce embolic events and morbidity, and reduce mortality in appropriate clinical scenarios [60-64]. The indications for surgical interventions are explained in Table 4 [65]. Cosyns et al. conducted a study across 40 countries from 2016 to 2018 with 3085 adult patients diagnosed with IE. Three hundred fifty-nine IE-cancer patients were compared with 2726 IE patients without cancer. It was found that the difference in the theoretical indication for cardiac surgery was minimum between the two groups. The study also found that cancer-IE patients were more frequently treated with bio-prosthetic valves than non-cancer IE patients (aortic bio-prosthesis - 76.3% vs. 56.2%, respectively; mitral bio-prosthesis 41.5% v.s 37.2%, respectively). Bio-prosthetic valve replacement was also preferred over mechanical valve replacement in cancer IE patients (aortic bio-prosthesis: 76.3% vs. mechanical 14.0%; mitral bio-prosthesis: 41.5% vs. mechanical 18.5%) [14].

**Table 4:** Echocardiographic features suggesting surgical intervention

ECHOCARDIOGRAPHIC FEATURES SUGGESTING SURGICAL INTERVENTION	
Vegetation	Persistent vegetation after systemic embolization.: Anterior mitral leaflet vegetation with size >10mm, ≥1 embolic event during the first two weeks of antimicrobial therapy, or ≥ two embolic events during or after antimicrobial treatment.
	Increase in vegetation size after four weeks of antimicrobial therapy
Valvular dysfunction	Mitral valve insufficiency with signs of ventricular failure or aortic valve insufficiency
	Heart failure in patients who are unresponsive to medical therapy.
	Ruptured or perforated wall
Perivalvular extension	Valvular dehiscence, rupture, or fistula
	New heart block
	Abscess or its extension despite appropriate antimicrobial therapy.

Although inconclusive, surgery in cancer patients for endocarditis seems to have an excellent prognostic effect compared to those who don't undergo surgery. Patients who were not surgically treated also had to discontinue their antitumor therapy due to the IE, worsening their life span [4]. Vivian et al. conducted a study that included patients with definite IE (International Collaboration on Endocarditis-PLUS, ICE-PLUS) from 2008 to 2012. The study concluded that patients with an indication for surgical intervention, who did undergo surgery were associated with a higher six-month survival than those who didn't receive surgery. It also found that those with higher and lower operative risks had similar outcomes when undergoing surgery. Still, those with a higher risk and who didn't undergo surgery had poorer outcomes [66]. Even though cancer patients have similar indications, only 50% of the patients undergo surgery. Infection is the most common indication for surgery in cancer-IE patients. But the most common reasons for denying surgery are the surgical risk itself and not cancer, death before surgery, or simply patient refusal [14]. The outcomes of surgery on cancer patients conducted by some studies are presented in Table 5.

**Table 5:** Outcomes of surgery as per included studies IE - Infective Endocarditis

REFERENCE	TIMELINE	TYPE OF STUDY	POPULATION	CONCLUSION
Cullen Grable, et al. (2021) [50].	2001-2006 and 2015-2018	Retrospective	56 patients with cancer and IE	Not associated with significant increase in death (HR 0.671; 95% CI 0.086 to 5.242; p=0.7036).
Ana Fernández-Cruz, et al. (2017) [8].	2008 to 2014	Prospectively included	161 patients diagnosed with IE	Both groups had similar outcomes.
Kyu Kim, et al. (2017) [18]	2011 to 2015	Retrospective cohort study	170 patients with IE	One-fifth of the IE patients with cancer underwent surgery, and the in-hospital mortality rate was 53.3%.

In the case of NBTE, indications for surgical interventions have not yet been established. Still, surgery can be considered in patients with benign or curable cancers or in those patients who have severe valvular dysfunction or recurrent embolic events beyond sufficient anticoagulation therapy [67].

Limitations

IE has multimodal pathophysiology in cancer patients owing to the plethora of malignancies and because of their specific characteristics makes them susceptible to the disease. This article has only broadly discussed the most common etiologies among all cancers. The intricate mechanisms in each type of cancer and their targeted therapies need to be discussed in detail with much more data to follow the evidence-based medical practice.

## Conclusions

Infective endocarditis and malignancies, both being different entities in their own categories, have an entangled relationship. This article clears out some of the lesser-known pathogenesis of IE in cancer patients, such as immunosuppression, *S. bovis* colonization, POE, and hypercoagulability. Cancer patients need to undergo various invasive procedures, especially during the diagnostic phase, which places them at risk of being exposed to various pathogens in their already immune-compromised state. The inherent nature of many malignancies is to facilitate the formation of thromboembolism, which should direct us to be wary of those specific cancers and keep IE in the back of our minds. Although surgical indications are similar in both cancer-IE and non-cancer IE, they are less often considered. However, they do have some proven benefits over those not undergoing surgical treatment. Various medical interventions are still being studied intensely, and existing protocol suggested treatment options depend upon the type of causative microorganism and treating the underlying cancer. Antibiotic prophylaxis is still debated if there is substantial value in preventing IE in cancer patients; hence, they are not yet recommended. The clinical significance of this review helps doctors deal with such patients in understanding how to be cautious and choose the appropriate mode of intervention based on the evidence available. Each cancer patient is different and can be classified according to their relevant risk factors and be screened for possible IE. We feel that there are still more unknowns in both diagnostic and treatment protocols for these conditions, which once determined could pave the way for better prognostic outcomes in such patients.
